# Impact of a CXCL12/CXCR4 Antagonist in Bleomycin (BLM) Induced Pulmonary Fibrosis and Carbon Tetrachloride (CCl4) Induced Hepatic Fibrosis in Mice

**DOI:** 10.1371/journal.pone.0151765

**Published:** 2016-03-21

**Authors:** Leola N. Chow, Petra Schreiner, Betina Y. Y. Ng, Bernard Lo, Michael R. Hughes, R. Wilder Scott, Vionarica Gusti, Samantha Lecour, Eric Simonson, Irina Manisali, Ingrid Barta, Kelly M. McNagny, Jason Crawford, Murray Webb, T. Michael Underhill

**Affiliations:** 1 The Centre for Drug Research and Development, Vancouver, British Columbia, Canada; 2 The Biomedical Research Centre, University of British Columbia, Vancouver, British Columbia, Canada; National Institutes of Health, UNITED STATES

## Abstract

Modulation of chemokine CXCL12 and its receptor CXCR4 has been implicated in attenuation of bleomycin (BLM)-induced pulmonary fibrosis and carbon tetrachloride (CCl_4_)-induced hepatic injury. In pulmonary fibrosis, published reports suggest that collagen production in the injured lung is derived from fibrocytes recruited from the circulation in response to release of pulmonary CXCL12. Conversely, in hepatic fibrosis, resident hepatic stellate cells (HSC), the key cell type in progression of fibrosis, upregulate CXCR4 expression in response to activation. Further, CXCL12 induces HSC proliferation and subsequent production of collagen I. In the current study, we evaluated AMD070, an orally bioavailable inhibitor of CXCL12/CXCR4 in alleviating BLM-induced pulmonary and CCl_4_-induced hepatic fibrosis in mice. Similar to other CXCR4 antagonists, treatment with AMD070 significantly increased leukocyte mobilization. However, in these two models of fibrosis, AMD070 had a negligible impact on extracellular matrix deposition. Interestingly, our results indicated that CXCL12/CXCR4 signaling has a role in improving mortality associated with BLM induced pulmonary injury, likely through dampening an early inflammatory response and/or vascular leakage. Together, these findings indicate that the CXCL12-CXCR4 signaling axis is not an effective target for reducing fibrosis.

## Introduction

### Hepatic fibrosis

Hepatic fibrosis is a pathological wound healing response to liver damage that is characterized by excess production and deposition of extracellular matrix (ECM) components [1–3]. Insults that can trigger a fibrotic response include viral infection, alcohol or drug toxicity, metabolic diseases and a variety of factors that induce an inflammation response in the liver [1, 4, 5]. The increased deposition of ECM and its altered composition lead to progressive functional deficits [6, 7]. Hepatic fibrosis and its end stage cirrhosis ranked 14^th^ and 10^th^ leading causes of death in the world and in developed countries respectively [8]. Unfortunately, this trend is expected to increase worldwide [8]. Hepatic fibrosis is reversible whereas cirrhosis, the end stage consequence of fibrosis, is generally not [2, 3]. Thus, it is important to identify therapy for hepatic fibrosis, as none currently exist [4, 6] and to prevent its progression to cirrhosis.

It has been established that hepatic stellate cells (HSCs) are the main cells contributing to the process of liver fibrogenesis [2, 4, 9]. HSC are fat and vitamin A storing cells in the body [10] but following liver injury, HSC become activated and undergo a morphological transition to myofibroblast-like cells [2, 11]. Activated HSCs produce an appreciable amount of ECM components [11]. Activation factors can include damaged hepatocytes, infiltrating inflammatory cells, endothelial cells, Kupffer cells (tissue marcrophages), changes in ECM composition and metabolites of toxic agents [2, 11].

### Pulmonary fibrosis

Idiopathic pulmonary fibrosis (IPF) is a chronic, irreversible and often fatal pulmonary disorder of unknown etiology and is characterized by progressive fibrosis of the lung parenchyma leading to scarring and loss of lung function [12]. IPF primarily occurs in older adults with a median survival time of 2–4 years after diagnosis [13, 14]. Prevalence in the United States has increased steadily from 202.2 cases per 100,000 people in 2001 to 494.5 cases per 100,000 people in 2011 [15]. Despite Phase 2 and 3 clinical trials indicating that pirfenidone was able to improve lung function in patients [16–18] there is still no current treatment for IPF as anti-inflammatory, anti-fibrotic and immunosuppressive therapies have proven ineffective [12, 19, 20]. Hence, there is an urgent need for an effective IPF therapy.

The fibrotic response in IPF appears to be driven by abnormally activated alveolar epithelial cells (AECs) which induce fibroblast proliferation, differentiation and recruitment [12]. Activated fibroblasts (myofibroblasts) secrete exaggerated amounts of ECM and destroy the architecture of the lung (reviewed in [12]). The origin of lung myofibroblasts remains a contentious issue. Recent lineage tracing studies based on a Foxd1-Cre line have shown that lung resident perivascular mesenchymal cells in addition to “lung fibroblasts” contribute to the lung myofibroblast population after bleomycin (BLM)-induced injury [21]. Circulating fibrocytes derived from bone marrow have also been reported to contribute to the myofibroblasts and type I collagen production in the lung [22–27]. However, recent studies have shown that bone marrow derived fibrocytes make a negligible contribution to type I collagen in lung fibrosis [28]**.**

### CXCR4/CXCL12 signaling and its putative role in lung and hepatic fibrosis

The 7-transmembrane G-protein coupled chemokine receptor, CXCR4 and its ligand CXCL12 (SDF-1α-stromal cell derived factor-1α) [29, 30] are involved in the homing of hematopoietic stem cells to the bone marrow, mobilization of stem cells from the bone marrow to the peripheral blood and injured tissues and act as a chemoattractant for different leukocyte populations [31–33]. CXCL12 is expressed in bile duct epithelial cells in normal human liver [34, 35] and its expression is upregulated in the liver and plasma of patients with advanced hepatic fibrosis relative to control patients [35]. Both human and murine HSCs express CXCR4 and its expression increases with HSC activation [36]. In particular, CXCR4/CXCL12 signaling has been shown to induce HSC proliferation and collagen I production [36]. Furthermore, liver sinusoidal endothelial cells have also been shown to express *Cxcr4* and another CXCL12 receptor, *Cxcr7* after hepatic injury [37] and to participate in liver regeneration and fibrosis.

In pulmonary fibrosis (PF), it was shown that circulating fibrocytes were increased in patients with stable IPF relative to controls and quantification of fibrocytes may even serve as an indicator of mortality in IPF patients [24]. Given the evidence that fibrocytes from the circulation and progenitor stem cells from the bone marrow maybe recruited to the lung during pulmonary fibrogenesis [22, 23, 25–27], it has been argued that the mobilization of these cells to the injured lung is likely in response to CXCL12 to mediate fibrosis [23, 25–27]. Specifically, inhibition of CXCR4/CXCL12 signaling with anti-CXCL12 antibody reduced recruitment of CD45^+^ ColI^+^ CXCR4^+^ fibrocytes in BLM exposed mice [25] and reduced lung fibrosis [23, 25–27].

### CXCR4/CXCL12 antagonist

There are a number of small molecule CXCR4 antagonists, initially generated as potential anti-HIV treatments or as hematopoietic stem cell mobilization agents [38]. The approved, i.v.-administered CXCR4 antagonist, plerixafor (AMD3100) [39], had been evaluated in a number of BLM-induced pulmonary fibrosis models [23, 26, 27] including one recently-retracted article [40]. In addition, AMD3100 had been used in models of hepatic injury with reported beneficial results in a rat model of acute liver failure [41] and a report of exacerbation in a murine model of chronic liver injury [42].

Recently, an orally bioavailable CXCR4 antagonist, AMD070, had been developed and had shown safety and proof-of-concept oral efficacy in a human clinical trial for HIV treatment [43, 44]. In the hope of resolving the apparently contradictory data for the effects of CXCR4/CXCL12 modulation in models of hepatic injury and evaluating whether CXCR4/CXCL12 could be a potential therapeutic target in fibrotic diseases, we tested the efficacy of AMD070 in a BLM induced murine model of PF and in a carbon tetrachloride (CCl_4_) induced murine model of hepatic fibrosis. Our results suggest that AMD070 was able to increase survival in BLM-induced PF but surprisingly, had no effect on lung fibrosis. Furthermore, AMD070 had no effect in a CCl_4_ induced murine model of hepatic fibrosis. Similar to its predecessor AMD3100, AMD070 showed a dose response for leukocytosis attributed to CXCR4 antagonism [45, 46]. In summary, our data suggest inhibition of the CXCR4/CXCL12 axis may alter the early inflammatory and vascular response to acute fibrosis but has no direct effect on the deposition of fibrotic matrix per se.

## Materials and Methods

All animal studies described in this article had complied with the Canadian Council on Animal Care guidelines and University of British Columbia Animal Care Committee. All animal studies had also been approved by the University of British Columbia Animal Care Committee.

Animals were housed in ventilated cages, maximum 5 per cage, in a 12-hour light/dark cycle and cages were changed once every 10 to 14 days. Animals received sterile food and water *ad libitum* and were handled aseptically. Animals were monitored at least twice daily with health monitor forms prior and post compound administration and any animal deeded to be at humane endpoint was euthanized. Criteria used for humane endpoint for the following experiments included one or more of the following: loss of >20% body weight, marked scruffed fur, hunched body, labored breathing, lack of response to stimulus and lethargic animal. To minimize animal suffering and distress, environmental enrichments such as shredded, crinkled brown paper for nest building and translucent, red polycarbonate house for shelter were provided. Isoflurane was also used as anesthetics when required as described below.

### Murine model of pulmonary fibrosis

PF was induced in six-weeks old female CD-1 mice purchased from Harlan. Animals were randomly divided into three groups (n = 10/group): PBS plus acetate buffer control, BLM plus acetate buffer and BLM plus AMD070. Some animals in the BLM plus acetate buffer and the BLM plus AMD070 groups reached a humane endpoint prior to day 22 (hence n = 4 and n = 9 respectively) and were euthanized.

On day 0, mice were anaesthetized with isoflurane and PF was induced by BLM administration. Specifically, BLM (C103610, Fresenius Kabi Canada, 2U/kg) in 40 μL PBS or vehicle control (PBS) was administrated by non-surgical endotracheal instillation with sterile disposable plastic loading pipette tips. The next day, AMD070 (Shanghai Haoyuan Chemexpress Co., 400 μg/mouse in 200 μL 30 mM acetate buffer) or 200 μL of 30 mM acetate buffer (pH 5) was administrated via oral gavage (PO) with a 20G feeding needle. AMD070 or acetate buffer vehicle was administrated daily for 21 consecutive days. Animals were euthanized with avertin overdose on day 22.

### Murine model of hepatic fibrosis

Hepatic fibrosis was induced in eleven week old female C57BL/6 mice from JAX Labs. Animals were randomly divided into three groups: oil plus vehicle (PBS) control, CCl_4_ plus vehicle (PBS) control and CCl_4_ plus AMD070. As some animals were euthanized prior to study completion because they had reached a humane endpoint, final group numbers were n = 9 for oil plus vehicle (PBS) control, n = 6 for CCl_4_ plus vehicle (PBS) control and n = 7 for CCl_4_ plus AMD070 groups. On day 0, mice received intraperitoneal (IP) injection of CCl_4_ (1 mL/kg) diluted in olive oil (1 part CCl_4_ and 3 parts olive oil hence 4 mL/kg of total volume) or olive oil control (4 mL/kg) twice a week for four consecutive weeks. Starting one day prior to the initial CCl_4_ treatment (day -1), dosing with either AMD070 reconstituted in PBS (50 mg/kg) or PBS vehicle control (10 mL/kg) was administered IP and continued 5 days/week for 4 weeks. Specifically, AMD070 or PBS treatments were not performed on the 2 days/week on which animals were given CCl_4_ (or olive oil vehicle control). One day after the last AMD070 or PBS treatment, animals were euthanized with CO_2_.

### Histology and percent fibrosis analysis of the lung and liver

Following perfusion with PBS, lungs were fixed in 4% paraformaldehyde at 4°C for 2 days. These were embedded in paraffin, sectioned at 1 mm apart and stained with hematoxylin-eosin (H&E) and Masson’s Trichrome stains. For quantitation of percent PF, entire lung and fibrotic areas were outlined. Percent fibrotic area was calculated by dividing fibrotic area (mm^2^) by total lung area (mm^2^) and multiplying by 100. For H&E stained slides, two histology slides/animal were selected from the central region of the lungs and analyzed whereas only one histology slide/animal was analyzed with Masson’s Trichrome stained slides. These analyses were performed with Image J and the analyst was blinded to sample identification.

Livers were perfused with PBS then 10% formalin and the left top lobe preserved in 10% neutral buffer formalin (NBF) for a week. The fixed liver was embedded in paraffin, six cross sections at 500 μm apart generated and stained with Picrosirius Red. For quantitation of percent hepatic fibrosis, fibrotic area (Picrosirius Red) was divided by total liver area and multiplying by 100. Three histology slides/animal were analyzed. The three slides corresponded to one slide from each of the alternative six cross sections. These analyses were performed with Olympus cellSens and the analyst was blinded to sample identity.

### RNA isolation and quantitative real-time qPCR

The top right lobe of livers were homogenized in 1 mL of Trizol (Invitrogen) and flash frozen in dry ice and stored in -80°C for RNA isolation to perform RT-qPCR. Total RNA was isolated and cDNA prepared using the High Capacity cDNA Reverse Transcription kit according to the manufacturer’s instructions (Life Technologies). RT-qPCR was carried out as previously described using the standard curve method [47]. The following primer/probe sets were used to detect: *Acta2* (IDT Assay–N007392.1), Probe- 5’-/56-FAM/TTACAGAGC/ZEN/CCAGAGCCATTGTCG/3IABkFQ/3’, Primer 1–5’-GTGAAGAGGAAGACAGCACAG-3’, Primer 2: 5’-GCCCATTCCAACCATTACTCC-3’; *Col1a1* (Life Technologies custom primer/probe mix), primer 1–5’-CTTCACCTACAGCACCCTTGTG, primer 2–5’- TTGGTGGTTTTGTATTCGATGACT, probe- 5’-FAM-ACACCGGAACTTGGG-MGBNFQ; *Tbp* (Life Technologies custom primer/probe mix), Primer 1–5’-AGAATAAGAGAGCCACGGACAACT, Primer 2–5’-TGGCTCCTGTGCACACCAT, Probe- 5’-FAM-CGTTGATTTTCAGTTCTGG-MGBNFQ; *Gapdh* (IDT Assay–Mm.PT.39a.1), probe- 5’-/56-FAM/TGCAAATGG/ZEN/CAGCCCTGGTG/3IABkFQ/3’, primer 1–5’-AATGGTGAAGGTCGGTGTG-3’, primer 2–5’-GTGGAGTCATACTGGAACATGTAG-3’.

### Serum aspartate aminotransferase (AST) activity

Five μL of serum was used with the AST Activity Assay Kit (Sigma MAK055) as per manufacturer’s instructions to determine serum AST levels.

### Pharmacokinetic (PK) studies and Hematology measurements

#### AMD070 PK studies via PO

Non fasted CD-1 female mice at six weeks of age from Harlan were sacrificed at 0.5, 1, 2, 3, 6, 24 and 48 hours (n = 3/time point) post administration of AMD070 reconstituted in 30 mM acetate buffer (pH 5) (200 or 400 μg/mouse). At each endpoint, mice were euthanized with CO_2_ and blood collected via cardiac puncture. An aliquot was set aside at room temperature for hematology measurements with remaining blood processed for plasma for drug concentration determination. Lungs were collected and immediately frozen on dry ice and stored at -70°C until determination of drug concentration.

#### AMD070 PK studies via IP injections

Non fasted C57BL/6 mice at 7 to 8 weeks of age from Harlan were sacrificed at 0.5, 1, 2, 3, 6, 24 and 48 hours (n = 3/time point) post administration of AMD070 (in 30 mM acetate buffer, pH 5) (400 μg/mouse). At each endpoint, animals were euthanized with CO_2_ and blood collected via cardiac puncture. An aliquot was set aside at room temperature for hematology measurements with remaining blood processed for plasma for drug concentration determination. Lungs and livers were also collected and immediately frozen on dry ice and stored at -70°C until determination of drug concentration.

#### AMD070 PK studies via SC (subcutaneous) injections

Non fasted C57BL/6 mice at 7 to 8 weeks of age from Harlan were sacrificed at 0.5, 1, 3, 24 and 48 hours (n = 3/time point) post administration of AMD070 (in 30 mM acetate buffer, pH 5) (400 μg/mouse). All preceding procedures were same as those for AMD070 PK studies via IP injections.

#### Bioanalysis of AMD070 in the plasma, lung and liver tissue

AMD070 concentrations in mouse plasma were determined using protein precipitation extraction followed by UPLC-MS/MS analysis. Briefly, 100 μL aliquots of plasma were transferred to individual wells of an Isolute PPT+ (Biotage) array plate containing 300 μL of 0.1% formic acid (FA) in acetonitrile (ACN). Following a few minutes of room temperature incubation, the samples were filtered by applying N_2_ (g) pressure using the Pressure+ 96 manifold (Biotage). 200 μL of sample filtrate was then transferred to a separate 96-well plate and evaporated to dryness using a Turbovap 96. The sample residue was then reconstituted into 200 μL of 0.1/5/95 FA/ACN/H_2_O (v/v/v) and analyzed using reverse phase chromatography (gradient elution) combined with multiple reaction monitoring acquisition (MRM). The mobile phases consisted of 0.1% FA in H_2_O and 0.1% FA in ACN.

AMD070 was extracted from lung and liver tissue by homogenization under basic conditions followed by liquid-liquid extraction in tert-butyl methyl ether (TBME) and ethyl acetate respectively. In summary, 50 mg of lung or liver tissue pieces were cut, weighed, and transferred to homogenization tubes. 25 μL of deionized water for lung and 10 μL for liver were added to each tube, followed by the addition of zirconium (Zr) homogenization beads. The samples were homogenized for approximately 20 seconds in the BeadBeater, followed by 1 minute of centrifugation at room temperature at 13,520 rcf. 200 μL of 1 N NaOH was then transferred to each tube, followed by a second homogenization/centrifugation cycle as described above. Next, 750 μL of TBME or ethyl acetate were added to each lung and liver tubes respectively followed by a third homogenization cycle (20 sec) and 10 minutes of centrifugation (room temperature, 13,520 rcf). 500 μL of TBME or ethyl acetate extracts were then transferred to corresponding wells on 96-well plates and evaporated to dryness. The dried residue was reconstituted into 0.1/5/95 TFA/ACN/H_2_O and analyzed using reverse phase chromatography (gradient elution) combined with MRM acquisition in positive electrospray mode (ES+). The mobile phases consisted of 0.1% TFA in H_2_O and 0.1% TFA in ACN.

## Results

### Pharmacokinetics and Pharmacodynamics of AMD070 administered PO

**Fig 1:**
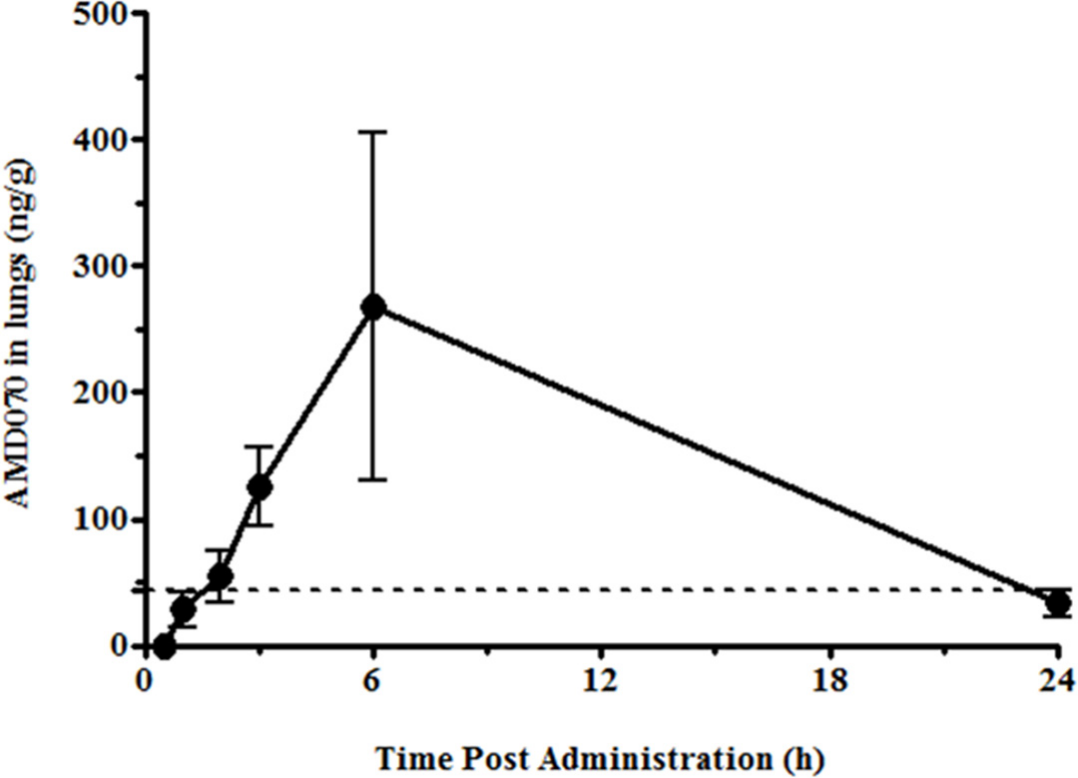
Pharmacokinetics of AMD070 in the lung of CD-1 mice. Concentration of AMD070 in the lungs of CD-1 mice at various times after PO administration at 400 μg/animal. Data represent the means (± SEM) of 3 mice. Horizontal dash line represents EC_90_ of 44ng/mL. (SEM, Standard error of mean).

Prior to the efficacy study of AMD070 administered by PO in the BLM-induced lung fibrosis model, we wished to confirm that AMD070 given by this route would accumulate in the lungs and in addition, had the anticipated effects on white blood cell (WBC) counts. CD-1 mice were given AMD070 by PO administration at a dose of either 200 or 400 μg/animal. Plasma and lung concentrations of AMD070, as well as complete blood cell counts were collected at various times after administration. Oral administration of AMD070 was associated with very low plasma concentrations of drug (data not shown) but a dose dependent accumulation of drug in the lungs was observed. Specifically, although mice given AMD070 at 200 μg/animal had detectable but not quantifiable drug levels, when administered at the higher dose of 400 μg/animal, reproducible drug concentrations in the lungs were observed with C_max_ concentrations of 267 ng/g observed 6 hours after PO (Fig 1). Importantly, the IC_50_ of AMD070 is 13 nM representing a concentration of approximately 4.5 ng/mL and a protein binding adjusted effective concentration (EC_90_) to achieve 90% effectiveness of 44ng/mL [48]. The lung concentrations achieved after oral administration of AMD070 (Fig 1) were significantly above both these thresholds for at least 21 of the 24 hours after dosing as determined by WinNonlin analysis.

**Fig 2:**
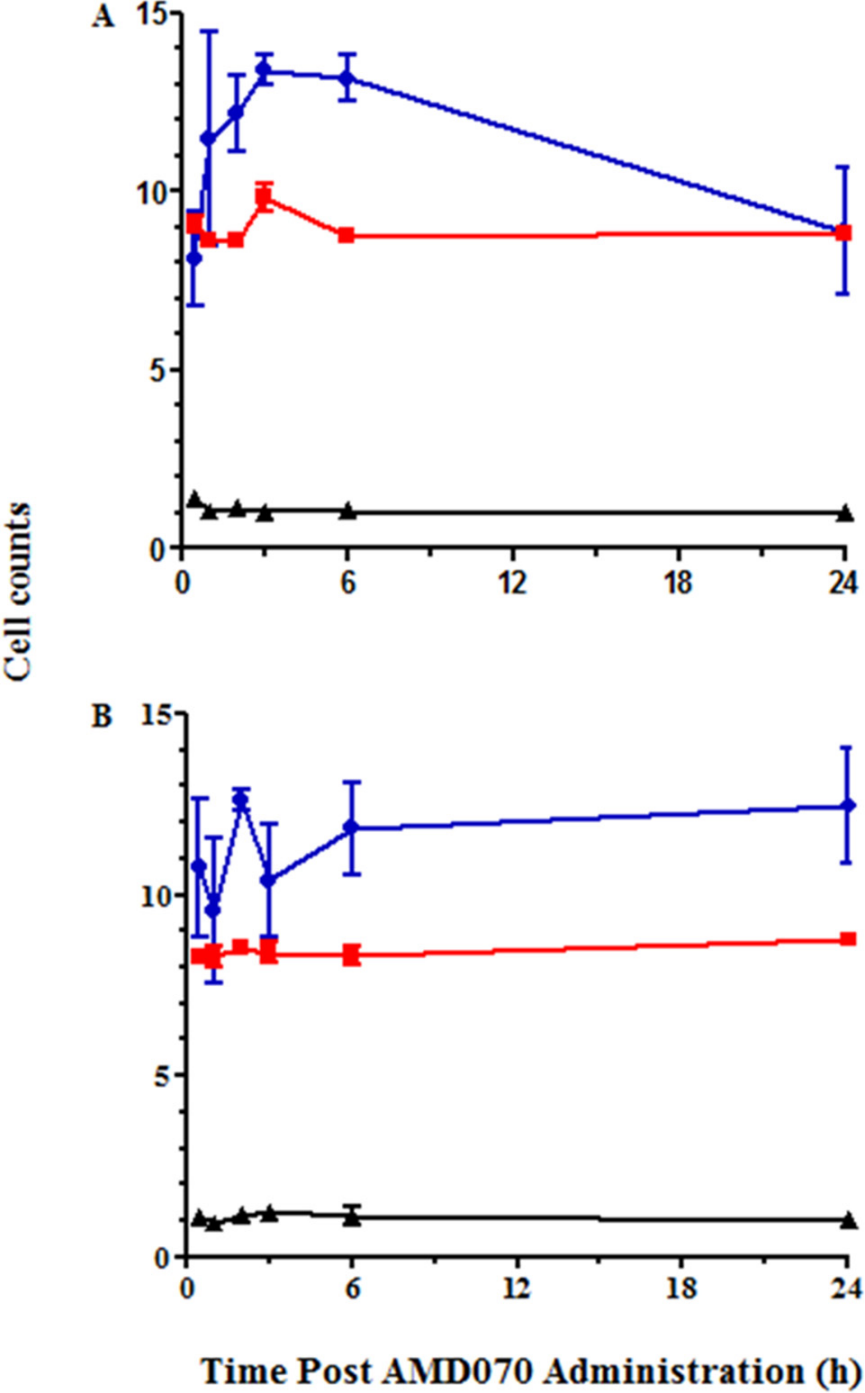
PO administration of AMD070 increased leukocyte mobilization. Cell counts in the blood of CD-1 mice at various times after the PO administration of AMD070 at either 200 (A) or 400 (B) μg/mouse. Data shown are WBCs (●; x 10^3^/μL), RBCs (■; x 10^6^/μL) and platelets (▲ x 10^6^/μL) and are the means (± SEM) of 3 animals.

**Fig 3:**
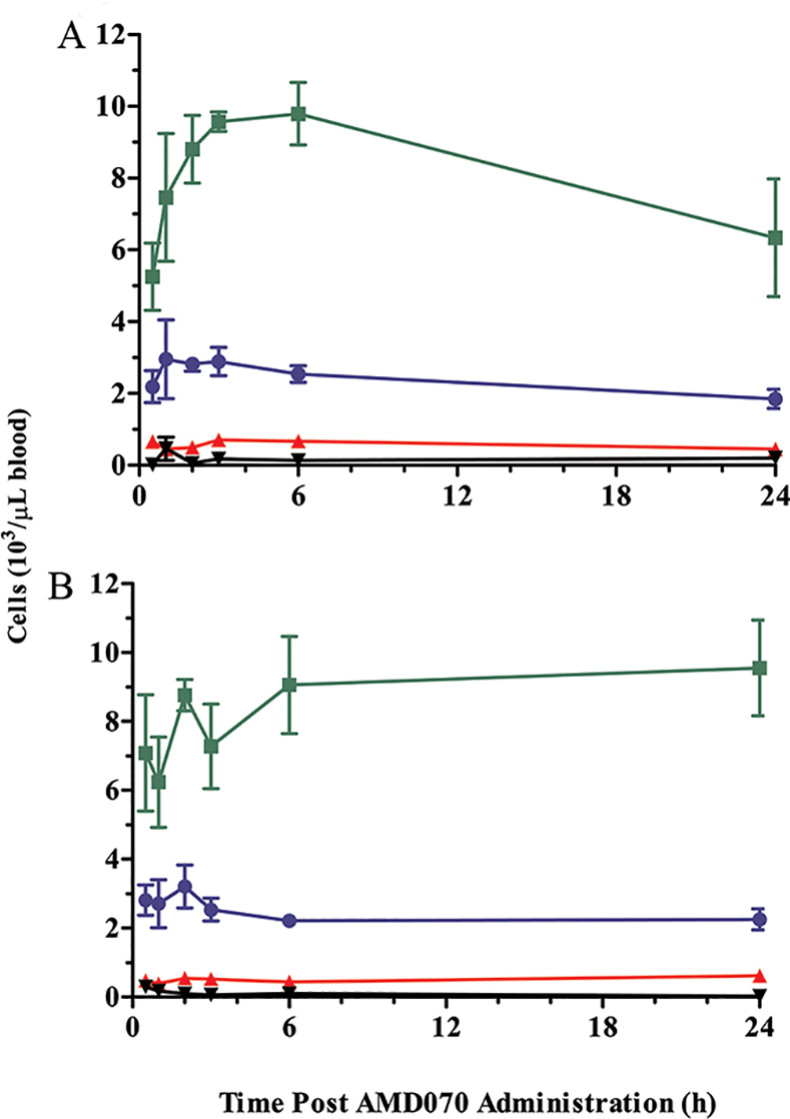
Leukocyte mobilization induced by AMD070 was the result of increase in lymphocytes. Differential cell counts in the blood of CD-1 mice at various times following PO administration of AMD070 at either 200 (A) or 400 (B) μg/mouse. Data are shown for lymphocytes (■), neutrophils (●), monocytes (▲) and eosinophils (▼) and are the means (± SEM) of 3 animals.

Consistent with these findings, we observed a dose-dependent effect of orally-administered AMD070 on WBC counts in these mice. Specifically, we observed a significant, transient increase in WBCs in mice dosed with AMD070 at 200 μg/mouse, with a T_max_ observed 3 hours following administration. At the 400 μg/mouse dose of AMD070, the WBC counts were increased and remained elevated for the full 24 hours of the study. In contrast, red blood cell (RBC) and platelet counts did not change after administration of AMD070 at either dose (Fig 2). White blood cell differentials are provided (Fig 3) and showed that the increase in WBC counts in whole blood shown in Fig 2 were the result of increased lymphocytes; we observed no increases in neutrophils, monocytes or eosinophils in these mice. These observations are consistent with literature reports on the effects of AMD070 on WBC mobilization [45].

### Efficacy of AMD070 in a murine model of pulmonary fibrosis

The IC_50_ and EC_90_ of AMD070 against CXCR4 were 4.5 ng/mL and 44 ng/mL respectively (Fig 1) [48], indicating that AMD070 concentrations in the lung were significantly greater than the IC_50_ and EC_90_ for the majority of the 24 hours after PO dosing. Based on these results, it was anticipated that oral administration of AMD070 at 400 μg/animal should achieve the needed systemic and tissue exposure to elicit a biological response in the BLM-induced model of PF. In addition, inhibition of CXCR4/CXCL12 had been shown to reduce lung fibrosis in a BLM induced murine model of PF [23, 26, 27].

**Fig 4:**
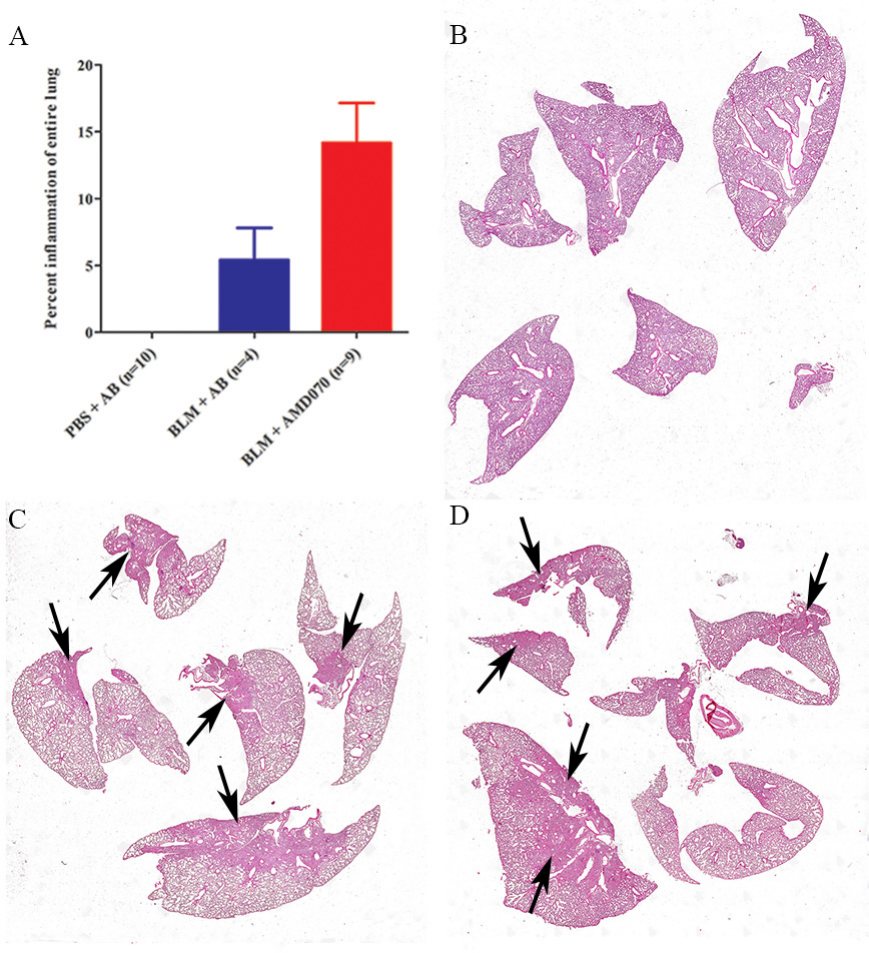
AMD070 did not alleviate BLM induced lung inflammation at end point as demonstrated by H & E stained lungs. Graph in (A) represents the means (± SEM) of percent surface area with high inflammatory cell infiltrate, as measured by H&E staining intensity. Representative H & E stained lungs of PBS plus acetate buffer (B), BLM plus acetate buffer (C) and BLM plus AMD070 (D) treated mice. Black arrows indicate areas with increased inflammatory cell infiltrate. AB indicates acetate buffer and this notation is used for the following Figs.

**Fig 5:**
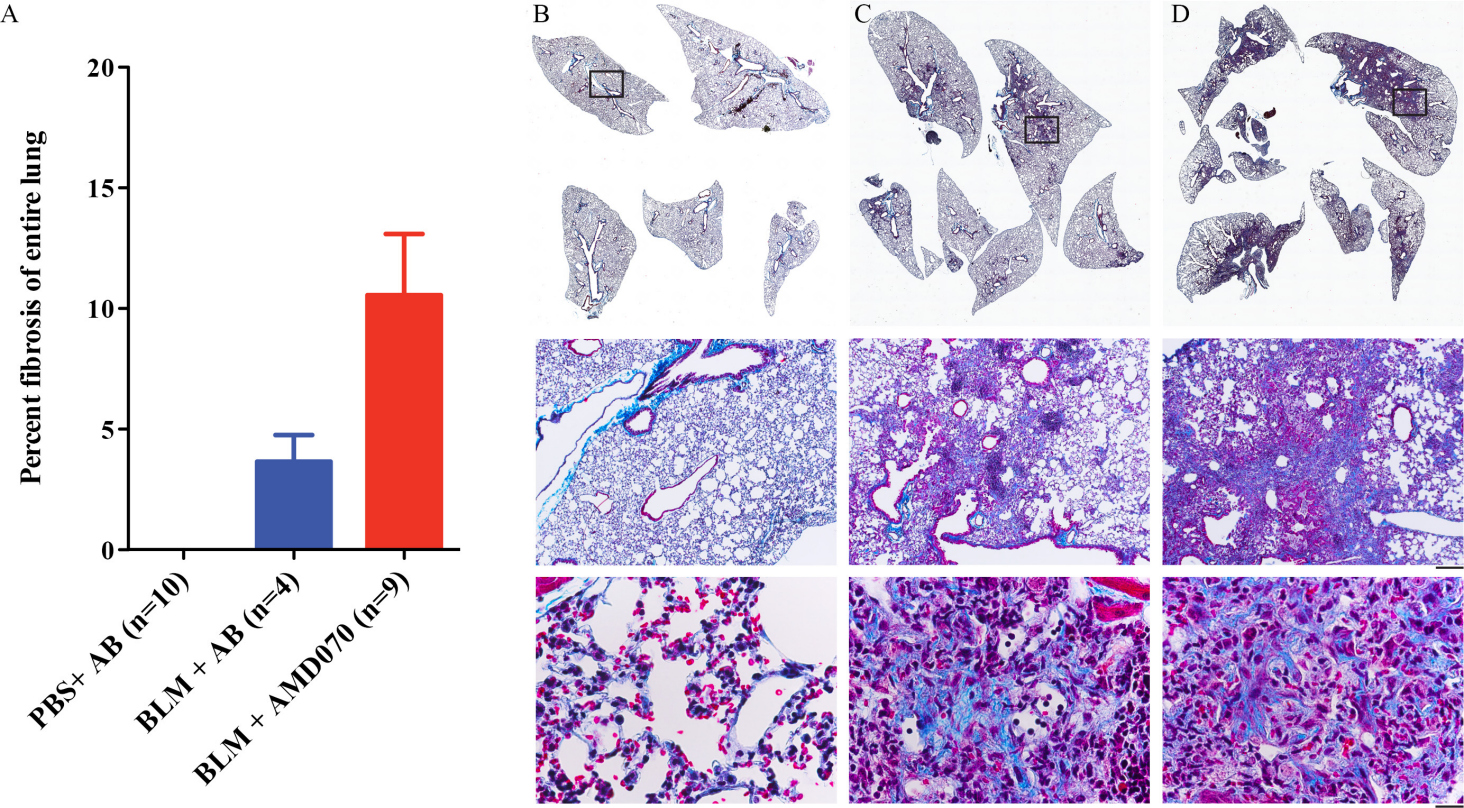
AMD070 did not alleviate BLM induced lung fibrosis as demonstrated by Masson’s Trichrome stained lungs. Graph in (A) represents the means (± SEM) of percent lung fibrosis. Representative Masson’s Trichrome stained lungs of PBS plus acetate buffer (B), BLM plus acetate buffer (C) and BLM plus AMD070 (D) treated mice. Note the intense cyan staining in the middle and lower panels indicative of collagen deposition in the lung parenchyma of BLM-treated mice. Bars represent 200 μm for the middle and 20 μM for the lower panel.

To determine whether AMD070 would have the anticipated effect, animals were randomly divided into three groups: PBS plus acetate buffer control, BLM plus acetate buffer and BLM plus AMD070. These groups were treated with BLM (or PBS vehicle control), AMD070 (or acetate buffer control) as detailed in Materials and Methods. Animals were euthanized one day after the last AMD070 treatment (Day 22). Treatment with AMD070 was not associated with reduction of lung inflammation and fibrosis in the surviving mice. H & E stained lungs showed an average of 5.4% (SEM ±2.4) lung inflammation in the BLM plus acetate buffer control; 14.2% (SEM ±3.0) in the BLM plus AMD070 and 0% (n = 10) in the PBS plus acetate buffer groups respectively (Fig 4). When lungs were stained with Masson’s Trichrome stains, there was an average value of 3.7% (SEM ± 1.1) lung fibrosis in the BLM plus acetate buffer control; 10.5% (SEM ±2.5) in the BLM plus AMD070 and 0% (n = 10) in the PBS plus acetate buffer groups respectively (Fig 5).

**Fig 6:**
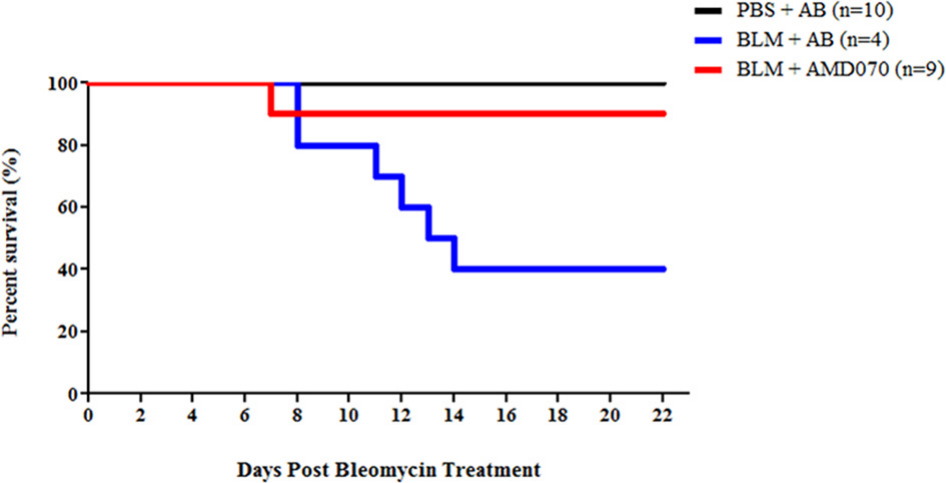
AMD070 alleviated BLM induced mortality. Kaplan Meier plot of animals in the PBS plus acetate buffer, BLM plus acetate buffer and BLM plus AMD070 groups.

In contrast to the absence of an effect of AMD070 on lung inflammation and fibrosis in BLM-treated mice, there was a significant effect of AMD070 on BLM induced mortality. Animals receiving AMD070 and BLM, showed 90% survival over the duration of the study (Fig 6). Those animals with BLM induced PF and treated with acetate buffer exhibited significant mortality between study days 8 to 14 with 60% of these animals requiring humane euthanasia (Fig 6). This mortality was almost entirely mitigated by daily gavage with AMD070; 90% of BLM treated animals receiving AMD070 survived to the completion of the study (Fig 6) indicating a very significant therapeutic benefit achieved by AMD070 in this disease model.

### Pharmacokinetics and Pharmacodynamics of AMD070 administered IP

**Fig 7:**
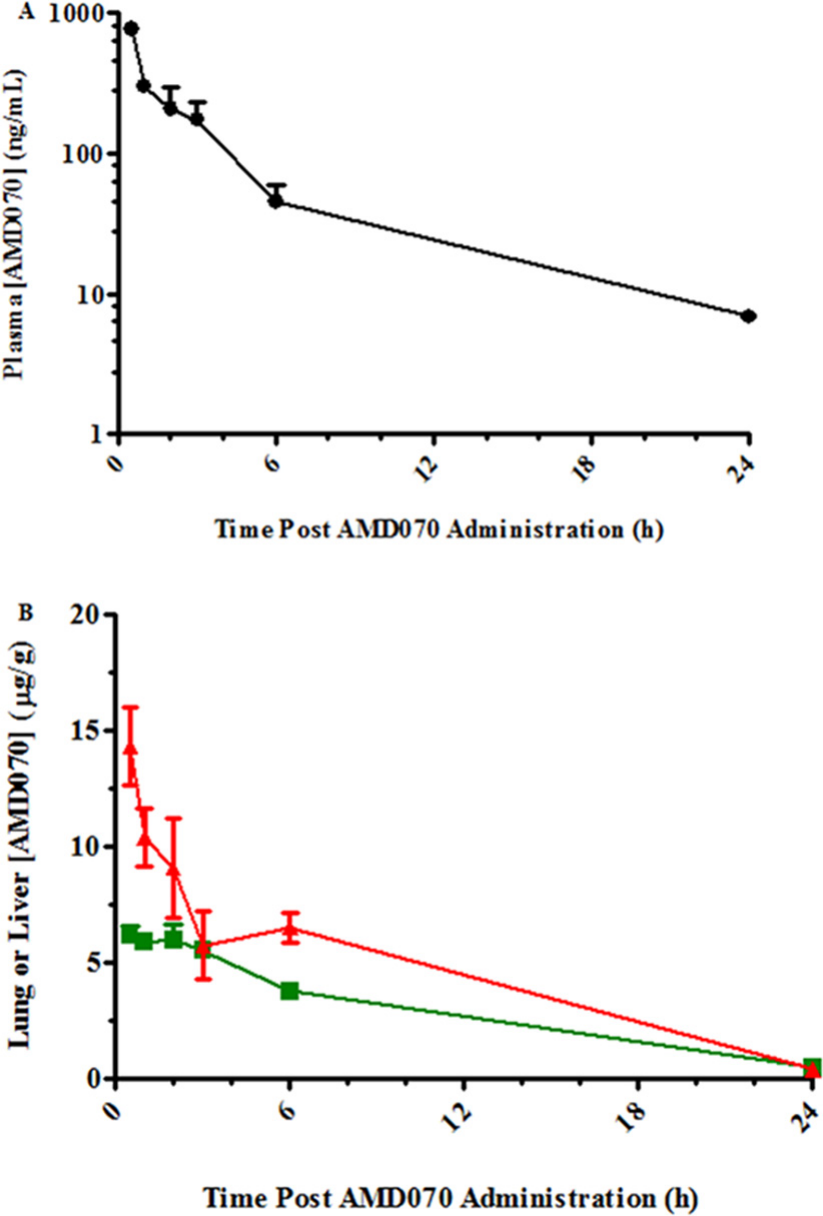
Pharmacokinetics of AMD070 in the lung, liver and plasma of CD-1 mice. Concentrations of AMD070 in the plasma (A) and in the liver (B; ▲) or lung (B; ■) of C57BL/6 mice at various times following IP administration. Values shown are mean (± SEM) of n = 4/time point except n = 3 for the 2 hour time point for the liver calculation.

As a prelude to an efficacy study of AMD070 in a murine model of hepatic fibrosis, we wished to alter the dosing route from PO to a route less likely to cause injury during repetitive dosing for 4 weeks. Therefore, a pharmacokinetic study was conducted to compare IP and SC routes of administration. After IP administration of AMD070, plasma concentrations of AMD070 peaked 30 minutes post administration at an average of 0.77 μg/mL of plasma and descending to an average of 0.045 μg/mL six hours post administration (Fig 7). In the lung, AMD070 concentration peaked 30 minutes post administration to an average of 6.14 μg/g of lung tissue and decreasing to an average of 3.74 μg/g six hours post administration (Fig 7). In the liver, AMD070 concentration peaked 30 minutes post administration to an average of 14.3 μg/g of liver tissue and decreasing to an average of 6.47 μg/g six hours post administration (Fig 7). When compared to plasma, lung and liver concentrations after SC administration of AMD070 (data not shown), the drug concentrations achieved using IP administration were somewhat higher than those achieved with SC administration. In summary, this showed substantial drug accumulation in the lung and liver post IP and SC administration of AMD070. The T_max_ (time to reach C_max_), C_max_ (maximum drug concentration) and AUC (area under the curve for all time points) values for both the SC and IP routes of administration are summarized in Table 1.

**Table 1:** Phoenix WinNonlin Noncompartmental analysis of AMD070 concentration in the plasma, lung and liver of C57BL/6 mice following IP or SC administration of AMD070 at 400 μg/mouse. Values for Cmax are provided as μg/mL for plasma and as μg/g for liver and lung. Values for AUC are provided as μg/mL for plasma and as μg/g for lung and liver.

	Plasma		Lung		Liver	
Routes	IP	SC	IP	SC	IP	SC
Tmax (h)	0.5	0.5	0.5	1.0	0.5	0.5
Cmax	0.77	0.70	6.14	5.65	14.29	5.76
AUC	1.24	0.97	75.74	80.76	112.33	77.18
Tissue AUC/Plasma AUC	-	-	61.18	83.34	90.74	79.65

T_max_ (time to reach C_max_)

C_max_ (maximum drug concentration)

AUC (area under the curve for all time point)

Tissue AUC/Plasma AUC (Tissue AUC divided by Plasma AUC)

**Fig 8:**
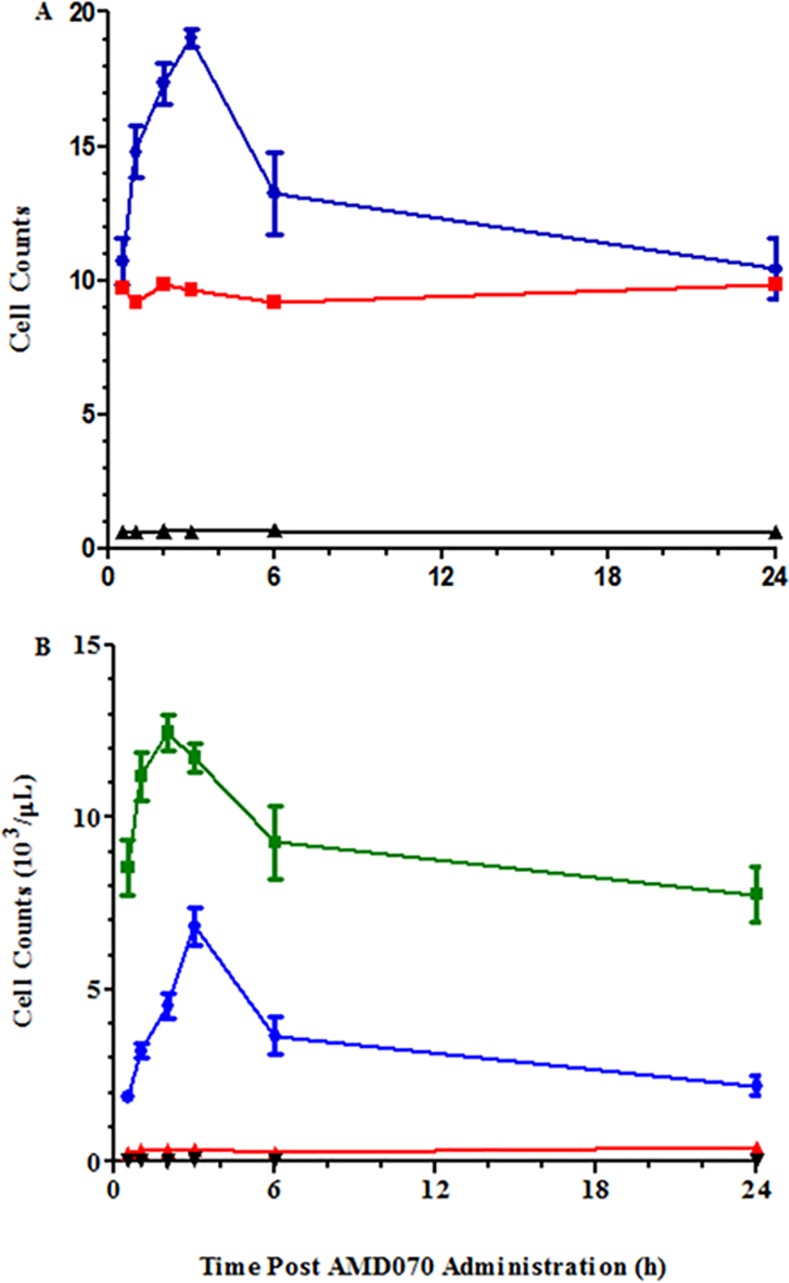
IP administration of AMD070 increased leukocyte mobilization. Cell counts in the blood of C57BL/6 mice at various times following the IP administration of AMD070 at 400 μg/mouse. A comparison of the numbers of WBCs(●; x 10^3^/μL), RBCs (■; x 10^6^/μL) and platelets (▲; x 10^6^/μL) are shown in panel (A) and are the means (± SEM) of 4 animals. Differential cell counts at various times are shown in panel (B) for lymphocytes (■), neutrophils (●), monocytes (▲) and eosinophils (▼) and are the means (± SEM) of 4 animals.

Consistent with these observations, there was a significant but transient increase in WBC counts observed with a T_max_ 3 hours following IP administration (Fig 8). In contrast, both RBC and platelet levels were unaffected by AMD070 administration (Fig 8). Differential analysis showed that the increase of WBC counts was largely due to increased lymphocytes and neutrophils (Fig 8). This analysis was also performed after SC administration of AMD070 (data not shown) and the qualitative trend was identical to that achieved with IP administration but the magnitude of these changes were lower in comparison to AMD070 given IP.

Based on these pharmacokinetic and pharmacodynamic observations of AMD070 given by IP and SC routes, IP administration was identified for use in the efficacy study of AMD070 in the murine model of hepatic fibrosis (below).

### Efficacy of AMD070 in a murine model of hepatic fibrosis

**Fig 9:**
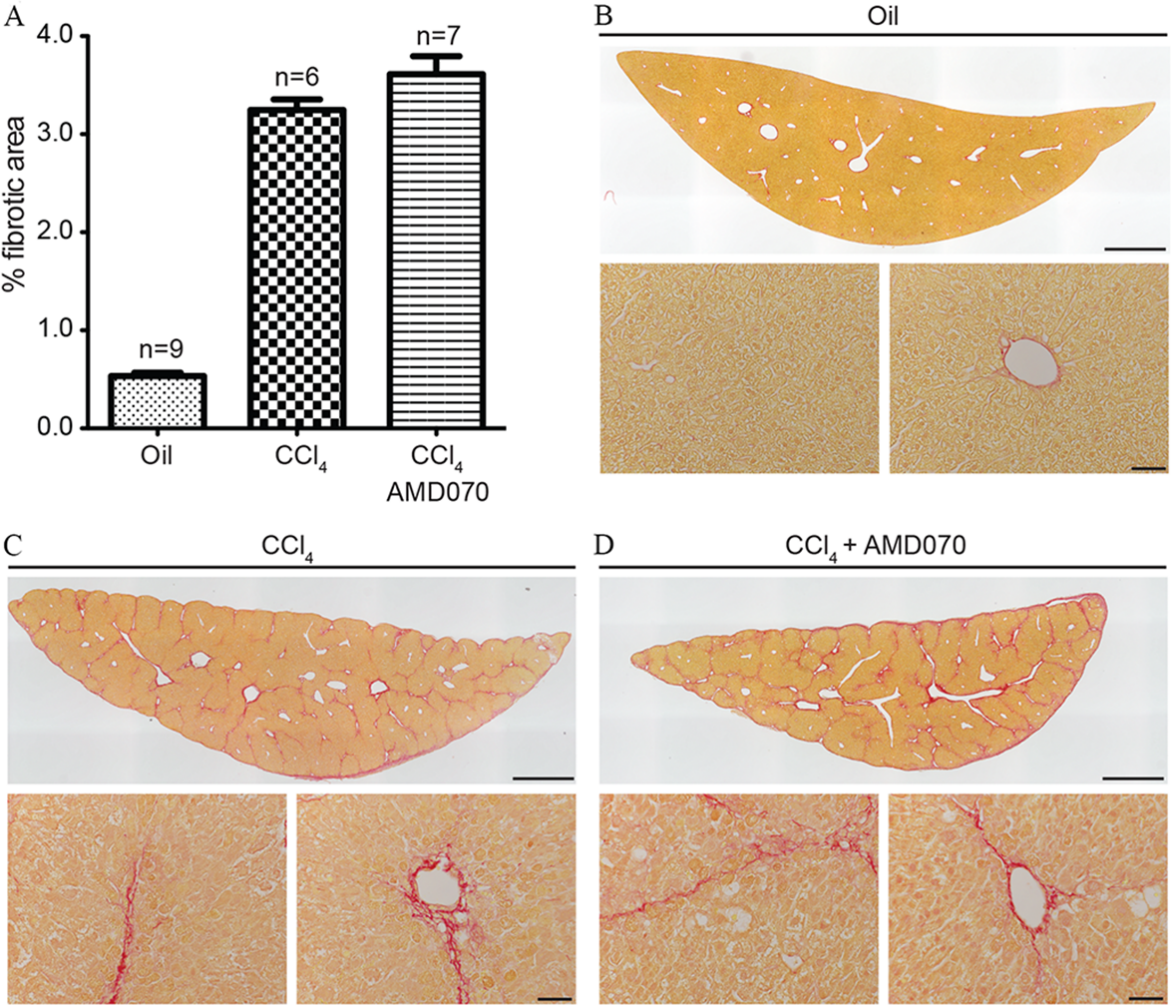
AMD070 had no effect on CCl_4_ induced liver fibrosis in C57BL/6 mice. Liver fibrosis was measured using Picosirius red and scored percent areas are shown in panel (A). Panels from each of the treatment groups contain representative Picrosirius Red stained left top lobe of livers from the oil plus PBS (B), CCl_4_ plus PBS (C) and CCl_4_ plus AMD070 (D) groups respectively. Bars represent 1 mm for the top image and 50 μm for the lower images in each panel.

Mice were randomly divided into three groups; oil plus vehicle (PBS) control, CCl_4_ plus vehicle (PBS) control and CCl_4_ plus AMD070 groups. After four weeks of treatment as detailed in Materials and Methods, livers were collected and stained with Picrosirius Red and analyzed for percent fibrosis. The top left lobe of the liver was analyzed for all animals. Treatment with AMD070 had no effect on percent liver fibrosis. There was a group average of 0.53%, 3.2% and 3.6% of Picrosirius red staining in the oil plus PBS, CCl_4_ plus PBS and CCl_4_ plus AMD070 groups respectively (Fig 9). There was also no difference in the mortality rate between these groups.

**Fig 10:**
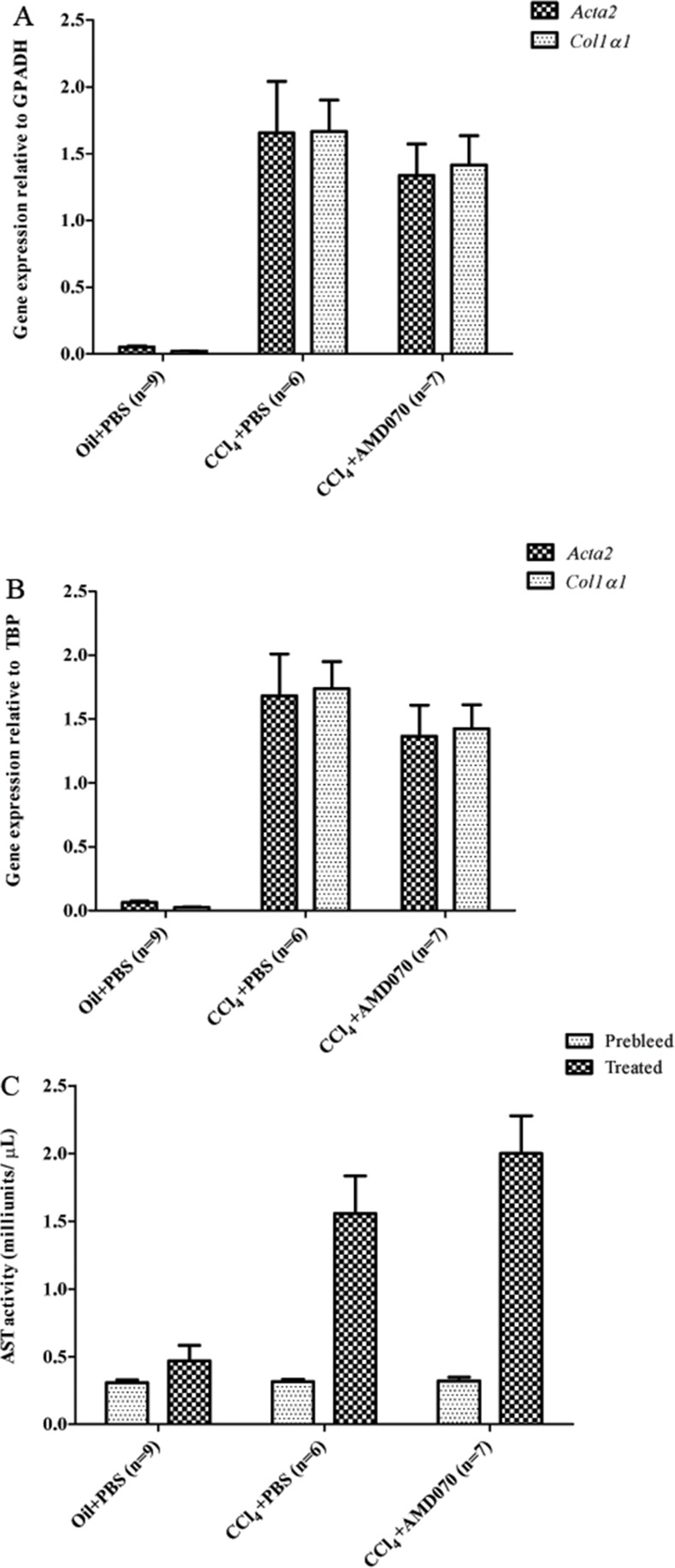
AMD070 had neither an effect on the *Acta2* and *Col1a1* transcription levels in the liver nor serum AST levels. The relative transcription levels of *Acta2* and *Col1a1* are shown relative to housekeeping genes *Gapdh* (A) and *Tbp* (B). Serum AST levels are shown in panel (C). Prebleed is serum collected prior to CCl_4_ and AMD070 treatment. Treated is serum collected one day after the last AMD070 or PBS treatment.

To determine whether AMD070 effects transcription of genes associated with a myofibroblast phenotype, RT-qPCR was performed on liver RNA collected one day after the last AMD070 treatment. The relative transcription levels of α smooth muscle actin (*αSma*, *Acta2*) and collagen α-1(I) chain (*Col1a1*) relative to housekeeping genes glyceraldehyde-3-phosphate dehydrogenase (*Gapdh*) and TATA binding protein (*Tbp*) were not different between the CCl_4_ plus PBS (n = 6) and CCl_4_ plus AMD070 groups (n = 7) (Fig 10A & 10B).

Serum AST levels are a marker for liver function and liver injury, and were measured prior to treatment and at the end of the experiment to both confirm CCl_4_ induced liver injury and to determine if there were beneficial effects associated with AMD070 treatment that were not observed in the fibrosis measurements. Prior to CCl_4_ or oil treatment, serum AST levels were similar between all three treatment groups. After treatment with CCl_4_ for 4 weeks, there was a significant increase in serum AST levels, confirming that liver injury was induced by this treatment (Fig 10C). However, those animals that received CCl_4_ and AMD070 had no difference in AST serum levels compared to the mice that received CCl_4_ and the vehicle control (Fig 10C). Together, these findings indicate that inhibition of CXCR4 has limited impact on hepatic fibrosis.

## Discussion

The overall goal of these studies was to evaluate whether CXCR4 was a potential target for therapeutic intervention in fibrotic diseases. Several studies [23, 26, 27] using CXCR4 antagonists have reported attenuated BLM-induced lung fibrosis in mice. Specifically, AMD3100, an antiviral compound belonging to the bicyclam derivatives that antagonize the CXCR4 receptor [49] was able to reduce lung fibrosis and fibrocyte infiltration into the lung [23, 26] in a murine model of BLM induced PF. In the present study, we have used AMD070, an orally bioavailable inhibitor of CXCR4 with improved tolerability [45] and pharmacokinetics, to evaluate the CXCL12/CXCR4 signaling axis in two different murine models of fibrosis. We have taken significant care to ensure that pharmacokinetics and tissue exposure of AMD070 was sufficient to elicit biological responses and have further confirmed the pharmacodynamic activity of AMD070 in each model by monitoring hematopoietic progenitor mobilization.

### Pulmonary fibrosis

AMD070 had no effect on the percent fibrosis in the lungs of the BLM-treated animals. As suggested earlier, this is inconsistent with studies by Makino et al. and Song et al. [23, 26]. It was suggested that bone marrow derived mesenchymal stem cells (BMDMSC) migrate out of the bone marrow and there is an increase of fibrocytes in the lung between 3 and 7 days post BLM injury [23, 26, 27]. It was reported that BLM treatment significantly induced chemotactic migration of BMDMSC to the lung 3 days post treatment and that this migration was inhibited by CXCR4 antagonists [26, 27]. Specifically, *in vivo*, AMD3100 decreased the number of fibrocytes in the lung at 3 and 7 days post BLM treatment [23, 26]. Increased expression of CXCR4 and CXCL12 in the lung was also observed beginning day 3 and up to day 21 after BLM treatment [26, 27]. Transplanted circulating human fibrocytes also migrated to fibrotic lungs in response to CXCR4/CXCL12 signaling in BLM induced murine PF [25]. Based on these and other observations, it has been proposed that CXCL12 chemoattracts BMDMSC and circulating fibrocytes to the lung via CXCR4/CXCL12 signaling during lung fibrosis [23, 25–27].

Previous reports demonstrated a role for CXCR4 in fibrosis, however, herein this was not observed. The absence of an effect of AMD070 cannot be attributed to insufficient drug accumulation in the lungs since we measured AMD070 accumulation in the lungs in significant excess of the CXCR4 IC_50_ and EC_90_ for 21 of the first 24 hours after dosing (Fig 1). Furthermore, AMD070 administration was associated with the expected dose-dependent increase in peripheral WBCs in these animals peaking at three hours after administration (Fig 2). This observation is consistent with AMD070’s ability to induce a dose related mobilization of bone marrow hematopoietic progenitors with a peak between two to three hours post dosing in human subjects [45].

In spite of the absence of an effect of AMD070 on BLM-induced fibrosis, there was a very striking effect on mortality in this model. Specifically, AMD070 treatment was associated with an increase in survival from 40% to 90% (Fig 6). In a rat model of BLM induced fibrosis, it was suggested that a “switch” between inflammation and the fibrotic phase occurred at around day 9 [50]. Inflammatory cytokine levels (IL-1α, IL-1β, IL6 and IFN-γ) increased rapidly by 3 days post BLM treatment and remain elevated up to day 9 [50]. Concurrently, there was an increase in collagen deposition starting at day 9, which was more pronounced on day 14 and 21 with a decline of the inflammatory cytokines on day 14 [50]. Similarly, it was found that 7 days post BLM treatment, increased vascular permeability and infiltration of neutrophil and lymphocytes into the lung were observed in mice [51, 52]. By 14 days post BLM administration, proliferation of fibroblasts and lung fibrosis were observed [51, 52]. In our study, mortality in the BLM group occurred between days 8 to 14, a period of vascular leakage and inflammation prior to the fibrotic phase and which was almost completely mitigated by AMD070 (Fig 6). This is consistent with the report that another potent CXCR4 antagonist, AMD3100 was able to alleviate mortality associated with BLM induced PF [53]. These data lead us to conclude that a role for the CXCL12/CXCR4 signaling axis in BLM-induced fibrosis is negligible. However, there may be a very important role for signaling via CXCR4 in the earlier inflammatory and vascular leakage phase of injury in this model. Thus, this may represent an opportunity for therapeutic intervention.

### Hepatic fibrosis

Since the effects of AMD070 on BLM induced fibrosis were uncertain, we tested the efficacy of this drug in a CCl_4_ induced murine model of hepatic fibrosis and AMD070 treatment by IP injection. As was observed in the studies described above in which AMD070 was given by PO, when administered by IP injection, significant levels of AMD070 were observed in the plasma, lungs and liver of the mice for 24 hours after injection (Fig 7). In addition, IP administration of AMD070 was also associated with the expected pharmacodynamic effects on WBC mobilization (Fig 8). As expected, CCl_4_ treatment induced clear evidence of liver fibrosis based on Picosirus red staining (Fig 9) and liver injury based on serum AST levels (Fig 10C). However, treatment with AMD070 had no effect on liver function based on the AST levels (Fig 10C) and the percent liver fibrosis was not different between the CCl_4_ plus PBS vs. CCl_4_ plus AMD070 groups (Fig 9). When liver RNA was evaluated for the transcript levels of *Acta2* and *Col1a1*, their relative abundance were not different between the CCl_4_ plus PBS and CCl_4_ plus AMD070 groups (Fig 10A & 10B). These data are inconsistent with the study by Saiman et al., which demonstrated that inhibiting CXCR4 with AMD3100 increased liver fibrosis and increased transcript levels of *Acta2* and *Col1a1*[42].

Activation of HSCs is known to play a major role in liver fibrogenesis [2, 9, 11]. It had been shown that CXCR4/CXCL12 signaling activates HSCs and induces their proliferation, and can lead to increased production of collagen I under fibrotic conditions [36]. Furthermore, recent studies have demonstrated that the majority of myofibroblasts following liver damage derive from “activated” HSCs [54]. AMD070 was delivered at the outset of the study and throughout the CCl_4_ treatment period, with a negligible impact on multiple markers of fibrosis. These observations indicate that the CXCL12/CXCR4 signaling axis has a limited role in liver fibrosis. It is possible that other mechanisms of HSC activation independent of CXCR4/CXCL12 signaling are operating as suggested by Saiman et al. [42]. Evidence for this comes from studies in which knockdown of CXCR4 only partially suppressed the proliferative response of HSC to CXCR4/CXCL12 signaling [42].

## Conclusion

Taken together, our results showed little evidence for a key pathological role of signaling via the CXCL12/CXCR4 axis in either the BLM-induced PF model or the CCl_4_-induced hepatic fibrosis model. Collectively, our findings suggest that CXCR4 represents a relatively poor therapeutic target (at least in lung and liver) for modulating the fibrotic response. Instead, they suggest a significant effect of the CXCR4 antagonist on survival in the BLM-induced PF model only during the early inflammatory and vascular leakage phase. Future work should focus on exploring the biological signaling occurring during these phases of injury and recovery following BLM induced lung injury.
